# Contributions of Gamma-Aminobutyric Acid (GABA) Produced by Lactic Acid Bacteria on Food Quality and Human Health: Current Applications and Future Prospects

**DOI:** 10.3390/foods13152437

**Published:** 2024-08-01

**Authors:** Mehmet Arif Icer, Buse Sarikaya, Emine Kocyigit, Büşra Atabilen, Menşure Nur Çelik, Raffaele Capasso, Duygu Ağagündüz, Ferenc Budán

**Affiliations:** 1 Department of Nutrition and Dietetics, Faculty of Health Sciences, Amasya University, Amasya 05100, Turkey; buse.sarikaya@amasya.edu.tr; 2 Department of Nutrition and Dietetics, Faculty of Health Sciences, Ordu University, Ordu 52000, Turkey; kocyigitem@gmail.com; 3 Department of Nutrition and Dietetics, Faculty of Health Sciences, Karamanoğlu Mehmetbey University, Karaman 70100, Turkey; busra.atbln@hotmail.com; 4 Department of Nutrition and Dietetics, Faculty of Health Sciences, Ondokuz Mayıs University, Samsun 55000, Turkey; dyt.mensurenurcelik@gmail.com; 5 Department of Agricultural Sciences, University of Naples Federico II, 80055 Portici, Italy; rafcapas@unina.it; 6 Department of Nutrition and Dietetics, Faculty of Health Sciences, Gazi University, Emek, Ankara 06490, Turkey; duyguturkozu@gazi.edu.tr; 7 Institute of Physiology, Medical School, University of Pécs, H-7624 Pécs, Hungary

**Keywords:** lactic acid bacteria, gamma-aminobutyric acid, food quality, human health

## Abstract

The need to increase food safety and improve human health has led to a worldwide increase in interest in gamma-aminobutyric acid (GABA), produced by lactic acid bacteria (LABs). GABA, produced from glutamic acid in a reaction catalyzed by glutamate decarboxylase (GAD), is a four-carbon, non-protein amino acid that is increasingly used in the food industry to improve the safety/quality of foods. In addition to the possible positive effects of GABA, called a postbiotic, on neuroprotection, improving sleep quality, alleviating depression and relieving pain, the various health benefits of GABA-enriched foods such as antidiabetic, antihypertension, and anti-inflammatory effects are also being investigated. For all these reasons, it is not surprising that efforts to identify LAB strains with a high GABA productivity and to increase GABA production from LABs through genetic engineering to increase GABA yield are accelerating. However, GABA’s contributions to food safety/quality and human health have not yet been fully discussed in the literature. Therefore, this current review highlights the synthesis and food applications of GABA produced from LABs, discusses its health benefits such as, for example, alleviating drug withdrawal syndromes and regulating obesity and overeating. Still, other potential food and drug interactions (among others) remain unanswered questions to be elucidated in the future. Hence, this review paves the way toward further studies.

## 1. Introduction

Humans need safe and nutritious food in their life. However, challenges like population growth, urbanization, climate change, and conflicts affect food safety and security, leading to significant food loss. The short shelf life foods adds to this problem [1]. The spoilage process makes food unsuitable for consumption. About one-third of the world’s food is lost due to spoilage or waste, leading to significant environmental and economic consequences [2]. Unwanted microorganisms can contaminate food throughout the production and supply chains (the production, processing, distribution, or preparation stages) [3]. Strategies to control spoilage-causing microorganisms and foodborne pathogens in food products involve managing intrinsic factors such as pH, water activity, NaCl content, and nutrient components, as well as extrinsic factors, including temperature, relative humidity, and the preservation methods required for microbial growth in the food product. Chemical compounds—synthetic and natural—and antimicrobials of biological origin can be utilized to manage pathogens and extend the shelf life of food products [4,5]. Lactic acid bacteria (LABs), their metabolites, or both, are often used to prevent the growth of undesirable microorganisms and improve food safety and quality [3]. LABs are a group of bacteria generally recognized as safe (GRAS), functioning as natural bio-protectants and health promoters [3,6]. They are known for their role in fermenting food and are being explored as a way to preserve food naturally [7,8]. They produce antimicrobial substances that can help prevent spoilage and the growth of foodborne pathogens [9,10]. Most LAB species including *Levilactobacillus brevis*, *Lacticaseibacillus paracasei*, *Lactiplantibacillus plantarum*, and *Lactococcus lactis* produce γ-amino butyric acid (GABA) via α-decarboxylation of glutamate by the enzymatic reaction of glutamate decarboxylase (GAD) [11,12], a pyridoxal 5′-phosphate (PLP-dependent enzyme) [11]. The demand for GABA production suitable for food applications has risen with its commercial utilization [13]. Although GABA is widely present in plants, animals, and microorganisms, its concentrations in plants are generally low [14]. Microorganisms serve as a significant source of GABA, with many microorganisms, including yeast, fungi, and bacteria, demonstrating the capacity to synthesize GABA [15,16]. It has been reported that *Lactococci* can synthesize significant amounts of GABA, but the highest-performing GABA producers have been reported among *Lactobacilli*, specifically *Levilactobacillus brevis*, *Lactobacillus delbreuckii* subsp. *bulgaricus*, *Lentilactobacillus buchneri*, *Limosilacobacillus fermentum*, *Lactobacillus helveticus*, *Lacticaseibacillus paracasei*, and *Lactiplantibacillus plantarum* [17]. Microbial fermentation is an effective process for GABA accumulation. LABs, in particular, are one of the most essential GABA producers due to their food-available and high GAD activity for GABA production [18]. 

With LABs being widely accepted as GRAS and having a high potential for application in the fermentation industry, GABA-producing LABs in the food industry have attracted great interest in recent years. Many GABA-producing LABs have been isolated from fermented foods and are used to produce natural health-oriented foods enriched with GABA [18]. 

The widespread use of GABA is attributed to the gradual elucidation of its physiological functions. GABA and its receptors have also been found in the peripheral nervous system, the endocrine system, and other non-neural organs, which are involved in oxidative metabolism [19]. The mechanism of GABA’s action on various diseases is mainly suggested by its presence in the central nervous system and the nerves around various organs, thus regulating human functions through nerve signal transmission and various receptors [20]. It is a potent pain reliever, beneficial for cardiovascular function, and treatment of various neurological diseases, including Parkinson’s disease, Huntington’s chorea, and Alzheimer’s disease [19,21]. GABA exhibits significant health benefits, including anti-hypertensive, anti-diabetic, and anti-inflammatory properties. Moreover, its potential anticancer effects, by stimulating cancer cell death and inhibiting growth, offer hope for its future applications in cancer treatment [13,22]. 

From the point of view of market consumption, GABA-rich foods are becoming increasingly popular due to the various physiological activities of GABA [23]. Currently, GABA-enriched foods include mostly grain-based staple foods, beverages, dairy products, and some snacks. The health claims of these GABA-rich products are mostly associated with relieving insomnia or improving sleep, lowering blood pressure, and relieving stress [24]. GABA impacts cognitive functions such as cognition, emotion, and memory and controls central nervous system activity. Therefore, it is necessary to draw attention to how important GABA function is in regulating neuronal activity and maintaining a healthy and functional neurological system [19]. It has also been reported that GABA-enriched foods have other health benefits, such as relieving stress and fatigue, hepatoprotective effects, and protection against cisplatin-induced nephrotoxicity [24].

This review aims to detail the process of obtaining GABA from LABs, the factors affecting this process, the use of GABA in the food sector, and its possible health benefits in light of the current literature. Given the significant role of GABA in the food sector and the growing body of evidence supporting its beneficial effects on health, this review will provide a comprehensive understanding of the process.

## 2. Biosynthesis of GABA by LABs

GABA is a non-protein amino acid produced mainly by plants, animals, and microorganisms [25] and has different functions depending on the producing organism [26]. Several LAB strains producing GABA have been isolated from traditional fermented foods such as cheese, kimchi, paocai, yogurt, and fermented soya beans [18]. In a recent systematic review, GABA-producing *Lactobacillus* species were compiled as *Levilactobacillus brevis*, *Lentilactobacillus buchneri*, *Lactobacillus delbreuckii* subsp. *bulgaricus*, *Limosilactobacillus fermentum*, *Lactobacillus helveticus*, *Lacticaseibacillus paracasei*, *Lactiplantibacillus plantarum*, *Lactococcus lactis*, etc. [18]. The GABA production capacity of different species is highly variable. *Levilactobacillus brevis* is able to produce higher amounts of GABA compared to other LAB species [27]. At the same time, various strains of a species also have marked differences in GABA productivity [27,28,29,30].

**Figure 1:**
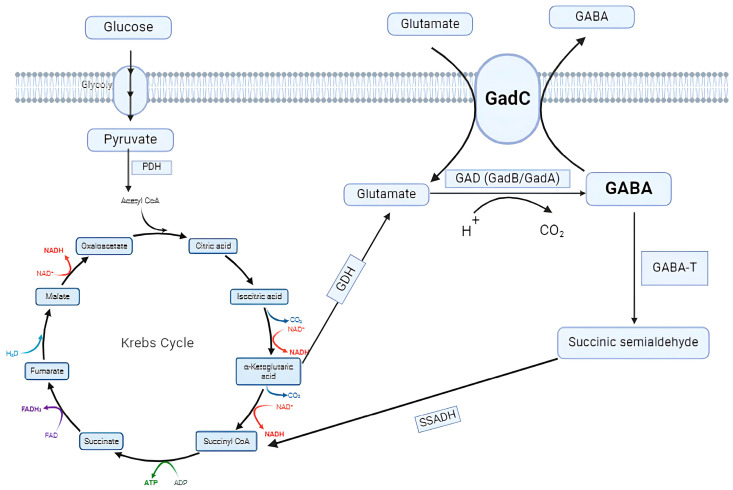
Metabolic pathway GABA production (Adapted from [18,46]). Abbreviations: PDH, pyruvate dehydrogenase; GDH, glutamate dehydrogenase; GAD, glutamate decarboxylase; GABA-T, GABA transaminase; SSADH, succinic semialdehyde dehydrogenase.

Some microorganisms use Putrescine (Puu) or GAD pathways for GABA biosynthesis [31]. The Puu pathway is a route used by some microorganisms (*Escherichia coli* [32] and *Aspergillus oryzae*, a fungus [33]) to obtain GABA [34,35]. In another pathway, the GAD pathway, GABA can be synthesized by a wide variety of microorganisms, including *Lactobacillus* spp. [36], *Escherichia coli* [37], *Listeria monocytogenes* [38], and *Aspergillus oryzae* [39]. Since this review focuses on GABA synthesis by LABs, the GAD pathway is detailed. The first step of the GAD pathway is carried out by an L-Glutamate (Glu)/GABA antiporter encoded by a gadC gene [40]. This antiporter pumps the precursor Glu or its monosodium glutamate (MSG) into the microorganism [41]. Subsequently, a PLP-dependent GAD enzyme catalyzes the conversion of the precursor to GABA, which is then transferred to the extracellular matrix by the action of the Glu/GABA antiporter [42,43]. L-glutamate precursor, a-ketoglutarate, is synthesized from glucose via the glycolysis pathway and part of the Krebs cycle and then converted to L-glutamate by L-glutamate dehydrogenase [18]. The GAD enzyme is encoded by a gadB gene that usually binds to PLP [37]. In most *Lactobacillus* strains (*Lacticaseibacillus rhamnosus*, *Lactiplantibacillus plantarum*, *Lacticaseibacillus casei*, and *Latilactobacillus sakei*), GAD is encoded by a gadB gene [44]. However, *Levilactobacillus brevis* also possesses a gadA, which presents a similar structure to the gadB gene. Although both genes play the same role in GAD expression, deletion of gadB is reported to be associated with a more pronounced decrease in GABA production than deletion of gadA [45]. The metabolic pathway GABA production is given in Figure 1.

### 2.1. Factors Affecting GABA Synthesis

Lactobacillus has attracted significant interest due to its many GABA-producing strains (e.g., *Lactiplantibacillus plantarum*, *Levilactobacillus brevis*, *Latilactobacillus sakeii*, *Lacticaseibacillus paracasei*, *Lactobacillus delbreuckii* subsp. *bulgaricus*, *Levilactobacillus zymae*, *Companilactobacillus futsaii*, *Lentilactobacillus buchneri*, *Lentilactobacillus parabuchneri*, *Levilactobacillus namurensis*, *Lacticaseibacillus rhamnosus*, and *Limosylactobacillus fermentum*) [13]. Depending on the natural environment of each *Lactobacillus* strain, different parameters influence the expression of GAD genes and, thus, GABA production [47]. These factors are explained below.

#### 2.1.1. pH and Temperature

pH and temperature are the main environmental factors that can modulate GAD gene expression [48]. The pH value is a key factor for GABA biosynthesis by LABs and affects the growth of the bacteria and GAD activity [22,45,49,50,51]. Changes in pH enhance GAD pathway activation, a key mechanism for maintaining cell homeostasis [27,52]. Some studies have shown that the initial pH of the fermentation medium affects GABA synthesis [22,51]. In a study analyzing how initial pH affects GABA production by *Lactiplantibacillus plantarum*, the best GABA concentration was found at pH 5.5, and it was reported that, at this pH, twice the amount of GABA obtained at pH 4.0 could be obtained [53]. In general, acid environment (as in Korean kimchi and Chinese paocai) has been reported to be beneficial for the growth of GABA-producing LABs [18]. Therefore, the optimal conditions for fermenting microorganisms vary according to the different properties of GADs, and the optimum pH is reported to be 3.5–5.0 [18]. Low pH must be maintained for effective GABA production [54,55]. It is also known that GAD activity is significantly lost at near-neutral pH (pH 7.0) [18].

At the same time, temperature also affects GABA production due to its relationship with GAD activation [53]. The researchers summarized the optimal temperatures and pH values for various *Lactobacillus* species. Accordingly, *Latilactobacillus sakei* showed the highest GAD activity at 55 °C and pH 5, while 40 °C and pH 4.5 were reported as the best values for *Lactiplantibacillus plantarum* GAD activity. In addition, different strains of *L. brevis* show optimum activity between 30 and 48 °C and at a pH of 4.2–5.2 [56,57]. The optimal temperatures of GADs ranged from 30 to 60 °C in different LAB species [18].

#### 2.1.2. Effect of Medium Composition

GAD activity is the key factor determining the GABA yield of a strain. Not only pH and temperature but also adding various media additives, such as L-glutamic acid and PLP, can modulate GABA synthesis. L-glutamic acid, the substrate of GAD, is an indispensable compound in the medium for the synthesis of GABA by LABs since LABs cannot synthesize sufficient L-glutamic acid for GABA production. Monosodium glutamate (MSG) is usually used in GABA production because it can produce L-glutamic acid by hydrolysis [18]. By increasing MSG, the aim is to stimulate GABA production of GAD via the GABA shunt pathway. At the same time, some researchers have shown that excessive MSG can inhibit cell growth and reduce GABA production. The optimal MSG concentrations for various microorganisms in GABA production are different [30,43,54]. The concentration of the Glu or MSG precursors strongly alters GABA synthesis [58]. In one study, the relationship between the amount of GABA produced *Lactiplantibacillus plantarum* and the effect of Glu concentration was measured in the range of 0–600 mM, and it was found that GABA production increased sharply until a 400 mM Glu concentration was reached [59]. Another study evaluating how different MSG concentrations affect GABA production by *Lactiplantibacillus plantarum* reported that the optimum Glu concentration to obtain the best GABA results was 20 g/L [53]. In a study in which a range of 0 to 400 mM MSG was used to evaluate the GABA yield of *Levilactobacillus brevis*, the best result was obtained at 270 mM [43]. Despite the effectiveness of the direct addition of Glu or MSG, alternatives were sought to reduce economic costs [26]. Woraharn et al. (2016) used the fungus *Hericium erinaceus* as a source of Glu combined with a co-culture of two *Lactobacillus* strains. *Levilactobacillus brevis* was used to hydrolyze L-glutamine to Glu using an L-glutaminase, and *L. fermentum* was added to convert this Glu to GABA. Another technique to promote the secretion of Glu without external support is co-cultivation with a microorganism that synthesizes Glu [60]. Yang et al. (2015) used a strain of *Corynebacterium glutamicum* to produce Glu, which was then converted to GABA by *Lactiplantibacillus plantarum* via the fermentation of cassava powder [61]. 

PLP can increase GAD activity by acting as a cofactor for the GAD enzyme. The effect of PLP varies according to the time of the addition of PLP. It was found that PLP can greatly promote the GABA production of *Lacticaseibacillus paracasei* at concentrations of 10 or 100 µM in the initial culture medium [55]. In addition to coenzyme PLP supplementation [62], other procedures, such as regulation of Tween-80 concentration [27] and the addition of metal ions, can be used to increase GAD activity [48].

Furthermore, adding different carbon and nitrogen sources can help improve bacterial metabolism and thus enhance GABA synthesis. Zareian et al. (2012) used glucose (carbon source) and nitrogen to enhance the bacterial production of Glu without any other supplementation [63]. However, the optimal carbon and nitrogen source varies depending on the *Lactobacillus* species. Several studies have shown that glucose is the most efficient carbon source for *Lactiplantibacillus plantarum* [64] and *Levilactobacillus brevis* [58]. Similarly, Zhao et al. (2015) reported that *Lentilactobacillus buchneri* produced higher amounts of GABA in the presence of xylose [65]. Yi Song and Yu Chui (2017) observed that *Lacticaseibacillus rhamnosus* synthesized high amounts of this amino acid using galactose [66].

#### 2.1.3. Effect of Cultivation Time

The point at which optimum GABA production was reached varied depending on the *Lactobacillus* strain used. In one study, the highest GABA yield was detected after 60 h of cultivation using *Lactiplantibacillus plantarum* [59], while another study reported a higher GABA yield at 35 h when using another *Lactiplantibacillus plantarum* strain [62]. A study on *Levilactobacillus brevis* reported that the highest amount of GABA was reached in 30 h [67].

### 2.2. Mechanisms and Techniques to Improve GABA Production

The GABA production capacity of strains is significantly affected by culture conditions. Numerous studies have been conducted to increase GABA yield by optimizing fermentation conditions, such as optimizing the initial pH of the culture medium, fermentation temperature, fermentation time, L-glutamic acid concentration, PLP, media additives, carbon source, nitrogen source, etc. [43,51,68].

Several LAB strains have shown potential for industrial GABA production. However, there is a need to improve the production efficiency of LAB-derived GABA. Several strategies have been used to improve GABA synthesis by LAB strains [69]. These strategies can be grouped under two headings: strategies dependent on modern biotechnology and traditional fermentation optimizations [70,71,72,73]. As a modern strategy, genetic improvement based on understanding cell physiology can effectively increase GABA production by LAB strains [74]. Conventional optimization has also proven to be an effective way to increase GABA production of LAB strains [65,75,76]. LABs often face various environmental stresses, including acid, cold, heat, drying, oxidative stress, etc., during fermentation and industry application [77,78]. In response to these challenges, LAB strains need good metabolic capabilities, strong physiological endurance, and environmental suitability [79,80,81]. Physiology-driven engineering has become an important way to increase the productivity of industrially applicable strains by improving their physiological performance [81].

Genetic engineering is an important strategy to improve GABA bioconversion and increase GABA yield through directed modulation of metabolic pathways. The direct approach is overexpression of the key enzyme GAD. Genes encoding GAD have been identified to be heterologously or homologously overexpressed in model LAB strains (*Latilactobacillus sakei*, *Lactiplantibacillus plantarum*, and so forth) [18,82,83]. A recombinant *C. glutamicum* was constructed by co-expression of two GAD genes (gadB1 and gadB2) from *L. brevis* Lb85. Compared to strains with a single expression of gadB1 or gadB2, this co-expressing strain increased GABA production more than twofold [82]. In addition to the overexpression of the GAD gene, glutamate in the GABA synthesis pathway, GABA antiporter gene gadC, and the regulatory gene gadR can also be used as a pathway for overproduction to increase GABA efficiency in the species [83]. 

The key to genetically modifying LABs is recombinant protein production gene therapy and genome engineering of the DNA molecules of plasmids used to deliver the genes of interest [84]. Since LABs have a thick peptide-glycan layer that acts as a barrier for transferring exogenous DNA into cells, the use of plasmids is often limited by transformation efficiency [85]. Additionally, factors such as low plasmid copy number, endonuclease activity within cells, and species-to-species variation limit the use of plasmids in LABs [86]. Alternatively, using genome engineering tools to insert the gene of interest into the LAB chromosome can increase the genetic stability of these constructs [87].

The Cre-lox system is another pathway for genetic recombination in LABs [88]. It offers flexibility and high recombination efficiency by allowing the deletion or insertion of a specific gene in any region of the bacterial chromosome [89]. There are limitations to the use of the Cre-lox system, such as iterative screening procedures, off-target effects, and high rates of false positive colonies causing genomic instability [90]. Alternatively, clustered regularly interspaced short palindromic repeats (CRISPR)-Cas systems, which make lethal double-strand breaks in the targeted region to eliminate false positive or wild-type colonies during screening, have been developed to provide a high-throughput screening and genome editing platform [91,92]. Rapid progress is being made in genetic engineering of LABs using recombination and CRISPR-based systems [84]. The most common genera used in the field of genetic engineering of LABs are *Lactococcus* and *Lactobacillus* [93,94].

However, the use of genetically modified LABs in food production is limited, due to concerns related to the dissemination of modified strains, plasmids, and recombinant genes, and especially due to a lack of public acceptance. Therefore, the use of genetically modified LABs is not approved and requires legal regulation [9]. 

Increased GABA biosynthesis efficiency and GABA productivity can also be achieved by inactivating the competing pathways of GABA production. The GABA aminotransferase enzyme gadT directs GABA to the Krebs cycle and causes GABA degradation. When gadB and gadC genes are co-overexpressed in the gadT mutant strain, the final GABA concentration is found to be increased [95].

A sufficient amount of precursor substance (L-glutamate) is needed for GABA production. However, since GABA-producing LABs cannot synthesize high concentrations of this compound naturally, exogenous L-glutamate must be supplied. Therefore, some L-glutamate recombinant strains have been developed to provide L-glutamate [96,97]. It was also found that GABA production was significantly increased by improving L-glutamate supplementation through deletion of the 2-oxoglutarate decarboxylase subunit gene odhA or the pyruvate carboxylase gene pyc [98].

In addition, using multiple microorganisms is currently popular in the fermentation industry as some substances produced by co-culture strains can enhance each other’s growth [99,100]. Co-fermentation with different strains is therefore considered a crucial and promising route for high yields of GABA [18].

## 3. Food Applications of GABA Derived from LABs

The production of foods and beverages fermented by LABs is becoming increasingly widespread because the metabolites produced as a result of their activities improve product quality and health consequences [101]. GABA is one of these metabolites that bacteria synthesize from the L-glutamate found in foods with the enzyme glutamate decarboxylase in order to increase their tolerance to acidic environments [12]. *Levilactobacillus brevis*, *Lentilactobacillus buchneri*, *Lactobacillus delbreuckii* subsp. *bulgaricus*, *Limosilactobacillus fermentum*, *Lactobacillus helveticus*, *Lacticaseibacillus paracasei*, and *Lactiplantibacillus plantarum* are important LABs for GABA production. Some strains of Streptococcus thermophilus and *Lactococcus lactis* are prominent in the production of GABA-rich dairy products. In recent years, it has been found that some species belonging to the *Enterococcus*, *Leuconostoc*, *Pediococcus*, and *Weissella* genera can also produce GABA [18]. The GABA production capacity of different species is highly variable. Compared to other LABs, it has been reported that *Levilactobacillus brevis* can produce high amounts of GABA (205 g/L). However, there may be marked differences in the GABA efficiency of various strains of a bacteria species [27]. Therefore, the use of strains with high GABA productivity as starter cultures in some fermented foods can be used as an alternative technique in the production of functional foods that offer significant health effects [102]. 

In the food industry, the production of functional foods enriched with GABA is becoming widespread. Examples of these functional foods include GABA-enriched beverages, such as Gabaron Tea, white tea, fruit juice; GABA-enriched dairy products, such as fermented milk, yogurt, cheese; GABA-enriched cereal-based products, such as brown rice, fermented oat, wheat-based sourdough, quinoa flakes; and GABA-enriched legumes and soy products, such as adzuki beans, black soybeans, tempeh, fermented soybeans, etc. [103,104]. Among these products Gabaron Tea, for example, was a common GABA-enriched functional beverage that was commercially produced in Japan in the 1980s and black raspberry juice enriched with GABA is included in the list of GRAS. GABA can also be used as a food additive in some foods such as chocolates, potato snacks, bread, and biscuits [104]. The advantage of using LAB fermentation in foods is its high enrichment effect and suitability for the mass production of GABA [105]. However, the problem with GABA production from LABs is that it requires a controlled fermentation process [102]. In addition, disadvantages of LAB fermentation include its high cost and strain safety issues [105].

### 3.1. Applications of GABA Produced by LABs in the Baking Industry and Cereal-Based Products

Plant foods are dietary sources of naturally occurring GABA. For example, while the GABA content in wheat is quite low and insignificant compared to other grain products (0.7 mg/100 g for wheat flour), the food with the highest GABA content was recorded as whole grain oat (57.1 mg/100 g). The GABA contents of different rice types also vary. The foods with the highest GABA content among pseudocereals are stated as Tartary Buckwheat and quinoa (10.34 mg/100 g and 7.8 mg/100 g, respectively) [106].

Since the amount of GABA naturally found in foods is low, the GABA content in cereal-based foods can be increased through the fermentation process using LABs. When a brown rice was fermented with 1 × 10^7^ CFU/mL LAB (*Lactiplantibacillus plantarum*, *Lacticaseibacillus casei*, *Limosilactobacillus fermentum*, and *Lacticaseibacillus rhamnosus*) at 36 °C for 48 h, the GABA content increased from 4.64 mg/g to 6.93 mg/g (49%) [107]. Although the amount of GABA naturally found in wheat is low (0.7 mg/100 g for wheat flour) [106], the GABA content was increased to 19.9 mg/g in wheat germ as a result of the fermentation with *Lactiplantibacillus plantarum* 299v [108]. Similarly, an increase in GABA content has been reported in breads prepared by fermenting with LABs [109,110,111] and in fermented products prepared using pseudocereals [70,112]. Table 1 summarizes the GABA content increase in cereal-based products fermented by LABs.

Applications increasing GABA content in cereal-based products should be considered in terms of their effects on product quality as well as their effects on health. The fermentation process with LABs is important in terms of improving taste, flavor, aroma, and texture in cereal-based and bakery industry products [113]. At the end of the fermentation process, an improvement in the volume, color, brightness, and taste of the bread product was demonstrated [109,111]. Fermentation plays a role in improving the bioavailability of micronutrients by providing optimal conditions for the enzymatic degradation of phytates. Improving protein digestibility causes an increase in the levels of free amino acids, especially lysine, methionine, and tryptophan [114]. Due to their antimicrobial effects, fermentation with LABs is also important in the detoxification of harmful components such as toxins [113]. Additionally, the production of gluten-free cereal-based fermented beverages may be a good option for people with celiac disease or gluten sensitivity [115].

**Table 1:** Effect of lactic acid fermentation on GABA content in cereal-based foods.

Cereal Based Foods	Lactic Acid Bacteria	GABA Content	Reference
Brown rice	*Lactiplantibacillus plantarum*, *Lacticaseibacillus casei*, *Limosilactobacillus fermentum* and *Lacticaseibacillus rhamnosus*	6.93 mg/g	[107]
Rice bran	*Lactiplantibacillus plantarum* EJ2014	19.8 g/L	[116]
Wheat germ	*Lactiplantibacillus plantarum* 299v	19.9 mg/g	[108]
Quinoa sourdough	*Levilactobacillus brevis* CRL2013	26.6 g/L	[70]
Fermented bread production by adding wheat bran to surplus bread	*Pediococcus pentosaceus* F01 *Levilactobacillus brevis* MRS4 *Lactiplantibacillus plantarum* H64 *Lactiplantibacillus plantarum* C48	148 mg/kg dough	[110]
Steamed breads	*Levilactobacillus* sp. LB-2	4.95 mg/g	[109]
Wheat germ bread	*Lactiplantibacillus plantarum*	Wheat flour bread (5.17 mg/100 g) Raw wheat germ bread (26.64 mg/100 g) Fermented wheat germ bread (28.42 mg/100 g)	[111]
Amaranth flour bread (%20)	*Levilactobacillus brevis* A7 *Lactobacillus farciminis* A11	26.9 mg/kg 39.0 mg/kg	[112]
Fermented beverage produced from brown rice milk	*Lactobacillus pentosus* 9D3	14.3 mg/100 mL	[115]

### 3.2. Applications of GABA Produced by LABs in Dairy Products

*Lactobacillus*, *Streptococcus*, *Leuconostoc*, *Pediococcus*, and *Lactococcus* are LABs that play a role in the fermentation of dairy products. During the fermentation process, various biochemical changes occur that increase food quality, such as the conversion of lactose to lactic acid, the release of fatty acids, improvements in sensory properties such as taste and texture, and the production of bioactive compounds [117]. In addition, the breakdown of proteins into casein and whey peptides and the increase in the shelf life of the dairy products are among the other positive results of the fermentation with LABs [118]. 

The use of probiotic LABs in dairy products is important in the production of functional foods that can reduce cholesterol and support the diet with GABA. Additionally, LABs can reduce oxidative stress by increasing the level of antioxidant components [119]. Animal studies have shown that the GABA in fermented dairy products may have anti-insomnia [120] and anti-diabetic [121] effects. Studies on fermented dairy products show that the use of more than one bacterial strain together increases the GABA content compared to a single bacterial strain [122,123,124]. In addition, the increase in GABA content continues during storage, and the sensory characteristics of fermented dairy products are better than the control group [125]. However, especially in cheeses, proteolysis can stimulate the release of free amino acids that can be converted to toxic biogenic amines. The accumulation of biogenic amines such as histamine and tyramine can have adverse effects on health. In a study evaluating the safety of starter cultures in cheese, it was reported that the *Levilactobacillus brevis* TAUL1567 strain could produce tyramine (193.15 μg/mL). Also, the *Lactococcus lactis* TAUL88 and TAUL8000 strains and the *Levilactobacillus brevis* TAUL1567 strain have been shown to be capable of producing putrescine [126]. Therefore, it is important to use starter cultures that do not produce biogenic amines in the fermented foods. The effect of LAB fermentation on GABA content in fermented dairy products is given in Table 2.

**Table 2:** Effect of lactic acid fermentation on GABA content in fermented dairy products.

Fermented Dairy Products	Lactic Acid Bacteria	GABA Content	Reference
Fermented milk	From a total of 94 LAB strains, *Lactococcus lactis* L-571 and L-572 showed the highest production	86.0 mg/L 86.2 mg/L	[124]
Fermented milk	*Lactococcus lactis* and *Lacticaseibacillus rhamnosus* *Lactococcus lactis* and *Lacticaseibacillus paracasei*	185.81 mg/L 319.72 mg/L	[122]
Fermented milk	*Enterococcus Faecium* MDM21 and *Lactococcus lactis* subsp. *lactis* BRM3.	136 mg/L	[123]
Fermented sheep’s milk	Commercial starter (*Streptococcus thermophilus* and *Lactobacillus delbrueckii* subsp. bulgaricus) *Lacticaseibacillus paracasei* Lb24 *Lacticaseibacillus paracasei* Lb41 *Lactiplantibacillus plantarum* Lb56	~150 mg/L ~170 mg/L 191.9 mg/L 197.9 mg/L (Values refer to after 28 days of storage)	[125]
Iranian traditional dairy products	*Lactococcus lactis* 311 *Lactococcus lactis* 491	0.395 mg/mL 0.179 mg/mL	[127]
Yogurt	Control *Levilactobacillus brevis* CGMCC1.5954	35.33 mg/100 mL 147.36 mg/100 mL	[128]
Yogurt	*Lacticaseibacillus paracasei* (supplemented with spirulina)	99.63 μg/mL	[129]
Kefir	*Lactobacillus* sp. Makhdzir Naser-1	3.82 mg/mL (Initial milk GABA content: 0.01 mg/mL)	[130]
Cheese	*Lactiplantibacillus plantarum* L10 and L11	11.30 mg/100 mL	[131]

### 3.3. Applications of GABA Produced by LABs in the Other Food Sources 

LABs are also widely used in the fermentation of meat products. LABs metabolize the proteins, lipids, and glycogen in meat into smaller molecules through enzyme systems and are responsible for the development of a special taste in the final product. In addition, the fermentation process improves the physical and chemical properties of meat products and increases antioxidant and antibacterial metabolites and nitrite breakdown [132]. During fermentation, the product is also enriched with GABA. Related studies have shown that LABs increase the amount of GABA in the Vietnamese traditional fermented meat product Nem Chua [133], fermented sausage [134], and fermented fish products [135,136].

Legumes are a source of natural prebiotic ingredients including oligosaccharides, resistant starch, polyphenols, and isoflavones. These compounds provide various important physiological benefits due to their anti-inflammatory and immune system regulation as well as anti-cancer properties [137]. In a study conducted on mice with depressive-like behavior, it was shown that the GABA content in fermented Adzuki bean sprouts increased serotonin and norepinephrine levels and improved social interaction [138]. However, applications for legumes are still limited due to the presence of undesirable compounds such as phytic acid and saponin and their unpleasant sensory qualities [139]. Fermentation of legumes with LABs is important in reducing undesirable nutritional components such as phytic acid and in developing a healthier and technologically adapted symbiotic product [137,139]. Protein solubility, water and oil retention capacity, emulsification, and gel formation properties can change during the fermentation process; thus, the technological properties of the products can be improved. In addition, fermentation with LABs is important in the degradation of aromatic components, reducing undesirable taste and allergenic properties [139]. By fermenting legumes, snacks and beverages with enhanced GABA, such as bread, pasta, and yogurt, can be produced [137]. The effect of lactic acid fermentation on GABA content in other food sources is shown in Table 3.

Similarly, fermentation of fruits and vegetables with LABs can have positive effects on health through the production of bioactive components [140]. In a study, it was reported that strawberry juice with enhanced GABA had an anti-inflammatory effect and reduced serum TNF-α and IL-6 levels in mice [141]. In another study, the GABA in fermented *Hovenia dulcis* extract was shown to have liver-protective properties in mice [142]. In addition to its health effects, fermentation also improves the quality of the foods. The fermentation extends the shelf life of products by reducing or inhibiting foodborne pathogens in fruits and vegetables [143]. Fruit juices fermented by LABs have higher viscosity, enhanced aroma with the production of new compounds, and increased stability of phenolic compounds through the production of organic acids [101]. Recently, the production of vegan fermented fruit and vegetable juices that can be easily used by individuals with lactose intolerance or allergies has been at the forefront. In a systematic review, it was stated that these products offer strong antimicrobial and antioxidant properties, high vitamin, total phenolic substance, amino acid, exopolysaccharide content, and unique sensory quality [144]. Fermentation of fruit and vegetable juices with LABs is also effective in increasing the GABA content of the products [145,146,147,148].

**Table 3:** Effect of lactic acid fermentation on GABA content in other food sources.

Fermented Meat Products	Lactic Acid Bacteria	GABA Content	Reference
Traditionally fermented meat (Nem chua)	*Lactiplantibacillus plantarum* VL1	1.568 mg/mL	[133]
Dry-fermented sausage	*Lactiplantibacillus plantarum* KS-3, KS-11, KS-17, KS-25, *Lactiplantibacillus plantarum* subsp. *plantarum* KS-12, *Pediococcus acidilactici* KS-20, *Weissella hellenica* KS-24, *Lactiplantibacillus pentosus* KS-27, *Latilactobacillus sakei* KS-30, KS-82	1.657 mM for *Lactiplantibacillus plantarum* KS-25	[134]
Fermented fish	INS-A2 INS-A4	20.0 mg/mL 18.8 mg/mL	[135]
Fermented fish sauce	*Pediococcus pentosaceus* MN12	27.9 mM	[136]
**Fermented legume products**			
Red lentils Green lentils	*Lactiplantibacillus plantarum* No. 122 *Lacticaseibacillus casei* No. 210 *Lactiplantibacillus plantarum* No. 122 *Lacticaseibacillus casei* No. 210	4.53 μmol/g 2.91 μmol/g 9.35 μmol/g 8.48 μmol/g	[149]
Fermented chickpea milk	*Lactiplantibacillus plantarum* M-6	0.537 mg/mL	[150]
Isoflavone-enriched soybean leaves	*Lactiplantibacillus plantarum* P1201 and *Levilactobacillus brevis* BMK184	Increased from 144.24 to 173.09 mg/100 g	[151]
Fermented soymilk	*Lactiplantibacillus plantarum* Lp3	3.74 mg/mL	[152]
Fermented soymilk hydrolysate	*Lactiplantibacillus plantarum* LMG6907	859 mg/L	[153]
Soy yogurt	*Lactobacillus delbrueckii* subsp. *latis* KFRI 01181 and *Lactiplantibacillus plantarum* KFRI 00144	0.455 mg/g	[154]
Yogurt-style snack produced with leguminosae flours	*Lactiplantibacillus plantarum* DSM33326 and *Levilactobacillus brevis* DSM33325	110.9 mg/L (Before fermentation: 90 mg/mL)	[155]
Soybean sprout yogurt-like product	*Levilactobacillus brevis* NPS-QW 145	2.302 g/L	[156]
**Fermented fruit and vegetable products**			
Cucumber	Not specified	Fresh 0.83 mM Acidified 0.56 mM Fermented 1.21 mM	[157]
Kimchi	Different LAB strains were evaluated: *Lactiplantibacillus plantarum* isolates *Levilactobacillus brevis* isolates	5.8 to 101.7 mM 8.5 to 88.6 mM	[158]
Kimchi	*Leuconostoc mesenteroides* K1501 *Leuconostoc mesenteroides* K1627	22.13 mM 22.81 mM	[159]
Tomato juice	*Lactiplantibacillus plantarum* KB1253	41.0 mM	[146]
Litchi Juice	*Levilactobacillus brevis* LBG-29 *Levilactobacillus brevis* LBG-24 *Levilactobacillus brevis* LBD–14	3.07 g/L 2.29 g/L 0.327 g/L	[145]
Litchi Juice	*Lactiplantibacillus plantarum* HU-C2W	3.92 g/L	[148]
Black grape juice	*Lactiplantibacillus plantarum plantarun* IBRC (10817)	117.33 ppm	[147]

### 3.4. Potential Adverse Effects of High GABA Intake 

The literature regarding the potential side effects of GABA produced by LAB fermentation is limited. It is known that some toxic components (such as biogenic amines) can be produced during lactic acid fermentation. Identification of strains that produce beneficial metabolites but do not increase toxic compounds may expand the use of these bacteria in the health and food industries [160]. On the other hand, the effects of GABA as a dietary supplement or as a naturally occurring ingredient in fermented milk or soy matrices were studied in clinical trials. Data at dosages up to 18 g/d for 4 days and in longer studies at intakes of 120 mg/d for 12 weeks indicated no significant side effects related to GABA. It is possible that using GABA concurrently with anti-hypertensive drugs could raise the risk of hypotension because GABA may cause a drop in blood pressure. Caution is advised for pregnant and lactating women since GABA can impact neurotransmitters and the endocrine system, which includes elevated levels of prolactin and growth hormone [23].

## 4. Human Health Benefits of GABA

**Figure 2:**
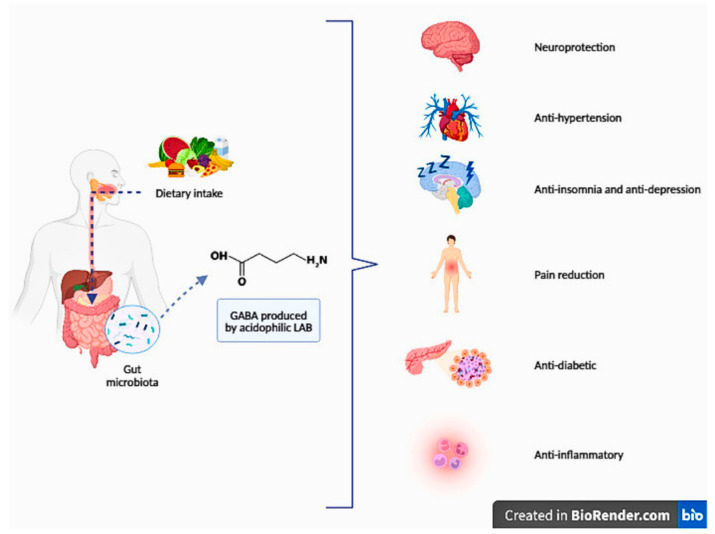
Potential health advantages of foods enriched with GABA (adapted from [24,162]).

In recent years, numerous studies have demonstrated the beneficial effects of GABA produced by LABs, referred to as a postbiotic, on neuroprotection, improvement of sleep quality, alleviation of depression, and pain relief (see Table 4). Foods enriched with GABA have been found to possess various health benefits, such as anti-diabetic, anti-hypertensive, and anti-inflammatory properties [24]. Despite its effects on different organs, GABA primarily exerts its influence through the brain–gut axis [161]. The positive effects of GABA on health are shown in Figure 2.

### 4.1. Neuroprotection

The inflammatory response to nerve tissue damage disrupts the balance of electrical activities between excitatory and inhibitory neurotransmitter systems in the brain by leading to the release of various inflammatory mediators such as reactive oxygen species (ROS), nitric oxide (NO), and cytokines [163,164]. This condition contributes to various neurological disorders, including epilepsy, Alzheimer’s, cerebrovascular diseases, multiple sclerosis, Parkinson’s, neuroinfections, and insomnia [165,166,167].

GABA is a significant inhibitory neurotransmitter in the nervous system and plays a critical role in transmitting information, neuronal development, and regulation of neurological disorders [168,169]. The impact of GABA on various diseases is attributed to its regulation of numerous functions through neuronal signal transmission and various receptors via its distribution in the central nervous system and peripheral organs [20]. Low GABA concentrations in the brain, imbalances in the GABAergic system, and alterations in GABA neurotransmitter activity, have been observed to lead to dysfunctions in ion transport functionality, synaptic connections, and modulation of the central nervous system [170,171]. Additionally, it has been noted that permanent damage to GABA function resulting from hypoxic–ischemic events during fetal development may lead to the emergence of learning and memory deficits [172].

The therapeutic effects of GABA as a dietary supplement have been extensively studied, showing its potential to enhance memory and cognitive functions by suppressing neurodegeneration [50,150,173,174,175,176,177,178,179]. It has been suggested that GABA supplements may reduce the severity of epileptic seizures and could be utilized for the prevention or mitigation of cerebral stroke damage [180,181]. However, in particular studies, the neuroprotective effect of GABA was not observed in cerebral ischemia [182,183]. In a study conducted on mice, significant neuroprotection could not be achieved when GABA transporters were inhibited following focal cerebral ischemia [183]. Similarly, consistent with the previous study, GABA did not exhibit a significant difference in functional or histological measurements following ischemia in the rat suture model, indicating no neuroprotective effect [182].

GABA produced by *Lentilactobacillus buchneri* isolated from kimchi protects against neurotoxic-induced cell death [50]. Additionally, GABA produced by another LAB, *Lactobacillus sakei* B216, isolated from kimchi, has improved long-term memory loss in cognitively impaired mice and increased the proliferation of in vitro neuroendocrine PC-12 cells [173]. Pre-germinated brown rice extract with enhanced levels of GABA has been observed to effectively inhibit apoptosis-associated DNA fragmentation and intracellular ROS formation, thereby significantly reducing the proliferation and apoptosis of human neuroblastoma cells [174]. Neuroprotective effects observed in mice fed with fermented rice flour containing 750.55 ± 26.03 mg GABA/100 g have been linked to increased activities of antioxidant enzymes superoxide dismutase and catalase in the cortex and cerebellum regions, along with a decrease in oxidative stress [175].

GABA-enriched fermented sea tangle (*Laminaria japonica*) fermented with *Levilactobacillus brevis* BJ20 has been shown to provide a protective effect against cognitive decline in dementia model mice and the elderly, potentially enhancing neuroplasticity [176,177]. Another study revealed that GABA-enriched fermented sea tangle with *Levilactobacillus brevis* BJ20 effectively increased neurotrophic factor levels associated with reduced risk of dementia and Alzheimer’s disease in middle-aged women [184]. GABA produced by *Lactiplantibacillus plantarum* from novel fermented chickpea milk has been reported to protect neuroendocrine PC-12 cells from MnCl_2_-induced damage and enhance cell viability, thus providing neuroprotective effects [150].

In conclusion, the neuroprotective effects of GABA may stem from its ability to improve long-term memory loss, support the proliferation of neuroendocrine cells, protect against neurotoxic-induced cell death, inhibit the proliferation and apoptosis of neuroblastoma cells, reduce ROS and oxidative stress levels, increase neuroplasticity and neurotrophic factor levels, and protect neuroendocrine cells.

### 4.2. Anti-Hypertension Activity

Hypertension, a condition where blood vessels are consistently under high pressure, typically arises from the narrowing or stiffening of the vessels [185]. Angiotensin-converting enzyme (ACE) plays a significant role in regulating blood pressure by converting angiotensin I to the potent vasoconstrictor angiotensin II; ACE inhibitors hinder this conversion, leading to vasodilation and consequently lowering blood pressure, thus proving effective in hypertension treatment [186,187,188]. It is noted that GABA found in foods fermented by LABs exhibits antihypertensive effects through ACE inhibitory activity, thereby playing a role in central blood pressure control in the cerebral renin-angiotensin system [20]. 

Skimmed milk fermented by the ND01 strain LAB demonstrated antihypertensive potential due to its high ACE inhibitor activities, with the *Lactobacillus helveticus* ND01 strain showing an ACE inhibitory activity of 67.18% [189]. GABA produced by *Lactococcus lactis* DIBCA2 and *Lactiplantibacillus plantarum* PU11 bacteria from fermented milk showed 0.70 ± 0.07 mg/mL ACE inhibitor activity [190]. Similarly, high ACE inhibitor activity has been observed in GABA produced by *Lactiplantibacillus plantarum* NTU 102 bacteria from fermented milk [191]. The production of 113.35 mg/L of GABA from skimmed milk by *Lactobacillus helveticus* has possible uses in the treatment of hypertension [187]. Additionally, 77.4 mg/kg of GABA obtained from milk fermentation with *Lactiplantibacillus plantarum* strain, when combined with other LABs, reaches a concentration of 144.5 mg/kg, thus providing an effective dosage for hypertensive effects [190]. Furthermore, yogurts containing GABA produced from *Lactobacillus helveticus* or *Lacticaseibacillus rhamnosus* have also shown antihypertensive effects [192].

The antihypertensive effects of GABA produced from fermented dairy products have been confirmed in rats [193,194] and humans [195,196]. In spontaneously hypertensive rats, after eight weeks of oral administration of GABA-enriched low-fat milk fermented by *Lactiplantibacillus plantarum* (80.6 mg/100 g), a decrease in systolic and diastolic blood pressure was observed [194]. Additionally, this effect of GABA from fermented milk was seen in another study involving both spontaneously hypertensive and normotensive rats [193]. In a study involving twenty-three adult men, daily consumption of 50 g of Cheddar cheese containing 16 mg of GABA prepared with *Lactococcus lactis* ssp. lactis strain over 12 weeks decreased 3.5 ± 2.8 mmHg in blood pressure and 5.5 ± 3.9 mmHg in systolic blood pressure [196]. Another randomized placebo-controlled study conducted in mildly hypertensive patients showed that supplementation of 10–12 mg of GABA in 100 mL of fermented milk significantly reduced blood pressure within 2 or 4 weeks [195]. 

It has been reported that GABA-enriched wheat-based fermented rice, fermented brown rice, and buckwheat exhibit a strong ACE inhibitory effect, with buckwheat’s maximum ACE inhibition percentage being 2.57 times higher than that of pure buckwheat [197,198,199]. Studies on GABA-enriched rice [199,200,201,202,203], idli [204], and purple sweet potatoes [205,206] have consistently shown antihypertensive effects in both humans and animals. It has been observed that GABA produced by *Lactiplantibacillus plantarum* MNZ from wheat-based fermented rice prevents the increase in blood pressure in spontaneously hypertensive rats. Additionally, a decrease in aortic endothelin-1 protein expression was observed in these rats [199]. It has been reported that supplementation of idli, a fermented rice and black lentil meal, significantly reduced systolic blood pressure in spontaneously hypertensive rats. The blood pressure-lowering effects of idli were attributed to reduced gene expressions of ET-1, HSP70, NF-κB, and iNOS in the aorta of spontaneously hypertensive rats [204]. In a randomized, double-blind, placebo-controlled clinical trial involving 39 mildly hypertensive adults, 150 g/day of GABA-enriched rice decreased morning blood pressure after the first week and between the sixth and eighth weeks compared to placebo rice [200]. GABA-enriched rice reduced blood pressure by approximately 20 mmHg in spontaneously hypertensive rats. However, it did not show a significant hypotensive effect in normotensive rats [201]. Similarly, the antihypertensive effect of GABA-rich brown rice has been demonstrated in spontaneously hypertensive rats [202,203]. Moreover, the blood pressure of patients with mild to moderate hypertension decreased significantly during daily consumption of 120 g of GABA-rich bread [207].

Research indicates that GABA derived from plant proteins such as beans, soybeans, and lentils exhibits high ACE inhibitor activity and has positive effects on hypertension [208,209,210,211]. Fermented soybeans with approximately 1.9 g/kg GABA produced by *Levilactobacillus brevis* were found to have higher ACE inhibitor activity than traditional soybeans [208]. Kimchi LABs fermented soybeans under optimized conditions, GABA content reached up to 1.3 mg/g and exhibited up to 43% ACE inhibitor activity [209]. An extract of 10.42 mg/g GABA obtained from fermented lentils showed a potent ACE inhibitor activity of 92% [211]. Significant systolic and diastolic blood pressure decreases were achieved in spontaneously hypertensive rats fed with GABA-enriched lentils [210]. On the other hand, the amount of GABA in foods needs to reach an effective dose for it to exhibit an antihypertensive effect. In a study evaluating the antihypertensive effects of eggplant, the GABA content in eggplants did not exhibit a hypotensive effect in spontaneously hypertensive rats [212].

GABA produced by purple sweet potato milk fermentation with LABs (*Lactococcus acidophilus* BCRC 14065, *Lactococcus delbrueckii* ssp. *lactis* BCRC 12256, and *Lactococcus gasseri* BCRC 14619) reduced both systolic and diastolic blood pressure. They showed positive effects on cardiac hypertrophy in spontaneously hypertensive rats [205,206]. Similarly, it has been shown that a GABA-rich tomato significantly reduces blood pressure in spontaneously hypertensive rats [213]. These findings confirm the ACE inhibitor activity of GABA found in fermented products and its ability to reduce blood pressure, supporting the consideration of fermented products as a potential alternative or adjunct therapy in hypertension management.

### 4.3. Management of Stress and Sleep

Insomnia, characterized by difficulty initiating sleep, poor sleep quality, and impaired daytime functioning, adversely affects individuals’ quality of life, mood, cognitive function, and health [214]. In individuals experiencing insomnia, GABA’s inhibitory function may be impaired, and reduced expression levels of GABA_A_ receptor α1 and α2 subunit mRNA may indicate sleep disorders [215].

Research on the effectiveness of GABA in reducing stress and improving sleep quality has yielded mixed results. While in a recent study on 19 sleep-disorder patients who took 700 mg/day GABA supplementation, the sleep score did not exhibit a statistically significant difference [216], others, mostly in animals, have demonstrated its potential to increase sleep duration [120,217,218,219], reduced sleep latency [120,219], and enhanced sleep quality both in animal and human studies [169,220]. Oral administration of GABA-rich fermented milk with *Levilactobacillus brevis* to mice induced with sodium pentobarbital or sodium barbital has increased sleep duration and reduced sleep latency [120]. Another study on mice showed that GABA derived from fermented rice seed extracts prevented caffeine-induced sleep disturbances, increased sleep duration, and mildly neutralized anxiety-like behaviors [217]. The alleviation of fatigue following the consumption of GABA-containing beverages has been demonstrated in both rats [221] and humans [222,223]. Oral administration of GABA Maoyecha tea extracts at a low dose (0.83 g/kg) for 30 days has significantly increased sleep duration and reduced sleep latency via GABAergic neurotransmission in mice induced with sodium pentobarbital [219]. Another study found that high-dose (3.33 g/kg) intake of GABA-rich black tea extracts for 15 days significantly increased sleep duration and proportion in mice induced with sodium pentobarbital. However, it did not significantly affect sleep latency [218].

Stress and anxiety are standard emotional states that impact people’s lives. Stress emerges as a response to external stimuli and is characterized by increased adrenergic activity [224]. Anxiety, on the other hand, is a personal response to prolonged or excessive stress, often defined as intense tension, worry, or anxiety associated with future adverse events [225,226]. Stress and anxiety have physical, psychological, and behavioral symptoms that can affect daily life and sometimes lead to pathological conditions. Therefore, maintaining an optimal stress level is essential for sustaining normal life processes [227]. Stress and anxiety are typically treated with lifestyle changes, psychotherapy, antidepressants, and anxiolytics [228]. 

The pathophysiology of anxiety is not entirely clear; however, research has shown that alterations in the GABA system are effective and that GABA improves mood [168,229]. Since the GABA receptor is the active site of anxiolytic drugs, GABA participates in depression and anxiety processes [230,231]. Therefore, it has been suggested that anxiety and depressive disorders can be treated with antidepressant drugs that regulate GABAergic transmission or GABA receptors. Additionally, it is noted that GABA tends to be less addictive compared to other antidepressants [230,231]. Furthermore, evidence suggests that oral GABA supplementation, when reaching concentrations that can produce biological effects in the brain, positively influences mood and sleep biology and plays a role in stress, anxiety, and depression [180,232,233]. Hence, it is recommended that GABA-containing foods and beverages could offer an alternative to pharmaceuticals in alleviating these conditions.

Animal studies have indicated reduced psychological and physical stress in animals fed with GABA-containing foods [219,234,235]. Rats fed with fermented black soybean milk containing GABA and subjected to forced swimming tests exhibited antidepressant-like effects without showing side effects such as loss of appetite or weight loss [234]. GABA-rich monascus, found on fermented rice or other grains, improved the levels of monoamines in the hippocampus of rats subjected to forced swimming tests, indicating an antidepressant effect [235]. In mice fed with GABA (3.43 mg/kg) produced by *Levilactobacillus brevis* J1 bacteria from fermented milk of adzuki bean sprouts for ten days, a decrease in mild depression-like symptoms was observed along with increased social interaction and mental activities [138]. 

A study on adults suggested that GABA increases alpha waves, decreases beta waves, and increases IgA levels under stressful conditions, implying that GABA can provide relaxation and reduce anxiety in stressful conditions [223]. Additionally, it is noted that GABA found in foods and beverages has a stress-reducing effect on acute and chronic stress in adults [236]. Supplementation with GABA-enriched yeast extract has been reported to alter human cortical excitation and inhibition balance [237]. Moreover, in more than 65% of 20 women consuming approximately 80 g of defatted rice germ enriched with GABA, the most common mental symptoms observed before menopause and in old age were significantly improved [238]. Another study showed that a single dose of chocolate containing 28 g of GABA produced by *Lactobacillus hilgardii* K-3 bacteria had a stress-reducing effect [239]. In contrast to previous studies, Konno et al. found that, in adults with sleep problems, the combination of GABA (700 g/day) and L-theanine (200 mg/day) significantly improved the Pittsburgh Sleep Quality Index score and Fitbit Charge 5 sleep improvement scores. Nevertheless, the sleep score did not exhibit a statistically significant difference. It has been reported that this might be attributed to a significant quantity of missing data about sleep duration and stage variables [216].

Studies conducted on animals and humans have demonstrated the effectiveness of GABA-fortified tea in alleviating stress, anxiety, and depression and relieving insomnia [240,241,242,243]. In studies on mice with post-ischemic stroke depression, both intraperitoneal injection and oral administration of GABA green tea via gavage have been shown to reduce depressive behaviors in mice. These reductions were determined by increased climbing and swimming times and decreased immobility time in forced swimming and tail suspension tests [241,242,243]. Additionally, it has been found that GABA green tea may increase GABAergic neurotransmission in the brains of mice [242]. In a study involving thirty young male participants, GABA-fortified tea was observed to reduce both acute and chronic stress and increase parasympathetic activity, thereby slowing down the heart rate. Moreover, it was noted that this effect was more pronounced in participants with higher levels of chronic stress [240]. In light of all these reports, it can be concluded that GABA has positive effects on sleep regulation, stress, and anxiety; therefore, consuming GABA-containing foods and beverages may benefit health and well-being.

### 4.4. Pain Reduction

Pain is a natural defense mechanism of the body that arises from nociceptors and includes the interaction of several neuroanatomical and neurochemical systems [244]. The International Association for the Study of Pain defines pain as “an unpleasant sensory and emotional experience related to actual or potential damage to body tissues”. Pain, which has a widespread impact on millions of individuals globally, has challenges in terms of treatment and can significantly influence one’s emotional well-being, social life, and profession. Pain is a multifaceted phenomenon encompassing several aspects, such as nociception, emotions, cognition, and social factors. It is a subjective experience that varies from person to person [245]. 

The etiology of pain is classified as nociceptive, inflammatory, or neuropathic. Pain arises from the interplay between receptors, neurotransmitters responsible for regulating the sense of pain, emotions associated with pain, and memories. Acute pain is a warning mechanism that protects us from tissue damage. On the other hand, chronic pain lasts for 3–6 months or more. It is a continuous pain linked to injuries, disorders, or diseases such as arthritis, gastrointestinal disorders, inflammatory bowel diseases, diabetes, and tumor growth. Chronic pain may result from nerve fiber damage, causing alterations in neurotransmitter function [246]. Research has shown that chronic pain problems are common in the general population. Based on the latest findings by the Centers for Disease Control and Prevention, it has been determined that a significant number of 51.6 million people in the United States are currently experiencing chronic pain [247]. A comprehensive survey of 52 nations revealed that the incidence of pain was documented at 28% [248]. The complete understanding of the molecular and cellular pathways that underlie persistent pathological pain remains incomplete. There is a need for clinically proven, well-tolerated, and effective treatment methods for chronic pain.

Recent research indicates that the gut microbiota significantly affects pain regulation [249,250]. The gut microbiota synthesizes neuroactive compounds such as GABA, tryptophan, and its metabolites, serotonin, and catecholamines. These compounds can communicate with the host through receptors on gut cells or neurocrine pathways [251]. Inhibiting the activity of ion channels in sensory neurons and blocking the transmission of C- and A-afferent fibers in the dorsal root ganglion (DRG) is regarded as an essential approach to decreasing hypersensitivity, increasing excitability, and alleviating the persistence of pain. Gut microbiota mediators, which include metabolites (SCFAs), neurotransmitters (glutamate, GABA, 5-HT), and pathogen-associated molecular patterns (PAMPs), control the excitability of nociceptive DRG neurons that act on pain-related receptors or ion channels (e.g., TRLs, TRP channels, ionotropic and metabotropic glutamate receptors, GABA receptors). They also lessen the activation of immune cells that secrete proinflammatory cytokines (TNF-α, IL-1, IL-6) and chemokines (CCL2, CXCL1) [249]. SCFAs can modulate pain sensitization by binding to their receptor FFAR2/3, which in turn regulates the synthesis of TNF-α, IL-2, IL-6, IL-10, and chemokines (such as C-C motif chemokine ligand 2, CCL2) by leukocytes [252]. The various metabolic pathways indicate that the gut microbiota could potentially significantly impact the regulation of neuronal excitability in the peripheral nervous system during chronic pain.

The gut microbiota can produce neurotransmitters influencing pain signaling [253]. LAB species, including *Lactobacillus* spp., *Lactococcus* spp., *Streptococcus* spp., *Bifidobacterium* spp., and *Bifidobacterium dentium*, produce GABA by using enzymes to remove a carboxyl group from glutamate [12,254]. The activation of the GABA_A_ receptor causes a chloride influx to cause hyperpolarization of the post-synaptic neuron. In contrast, GABA_B_ receptor activation decreases the likelihood of presynaptic neurons to release neurotransmitters, especially glutamate [255,256]. This inhibits the triggering of a neuron’s action potential and the release of synaptic vesicles. Gabapentin, a structural analog of GABA, has been used in medical practice for many years to alleviate thermal and mechanical pain [257]. Gabapentin and pregabalin have shown analgesic properties in individuals with pancreatitis, irritable bowel syndrome (IBS), and inflammatory bowel disorders. Also, they can alleviate abdominal wall discomfort and concomitant fibromyalgia [258,259,260]. Metagenomic research has revealed decreased *Lactobacillus* and *Bifidobacterium* in individuals suffering from visceral hyperalgesia and IBS [261,262]. In vivo studies have demonstrated that probiotics, including strains from the *Lactobacillus* and *Bifidobacterium* families, have a positive effect on lowering visceral pain [263,264]. *Lactobacillus* and *Bifidobacterium* are species that express the enzyme glutamate decarboxylase β (GadB), which decarboxylates glutamate to GABA [265,266]. GABA is capable of relieving pain. However, more research is required to determine if certain types of microbes can make GABA or similar molecules, as well as what effects this signaling has on the development and relief of chronic pain.

### 4.5. Modulation of Glucose Homeostasis

Diabetes mellitus (DM) is a chronic, endocrine, and metabolic condition characterized by elevated glucose levels and linked to the disruption of carbohydrate metabolism, either due to insufficient insulin production or a failure of the utilization of insulin [267]. GABA and GABA-enriched products can potentially be valuable therapeutic agents for controlling impaired glucose homeostasis [268].

GABA may play a crucial role in the pancreatic islet by regulating hormone secretions, suppressing the immune response, enhancing the survival of β cells, and facilitating the conversion of pancreatic α cells into β cells [269]. Furthermore, GABA has demonstrated its involvement in the control of insulin and glucagon secretions and its role in protecting and regenerating β-cells and promoting neogenesis. GABA suppresses immunological activation and inflammation in individuals with diabetes mellitus, resulting in the management of glucose homeostasis and a decrease in diabetic complications. According to in vivo studies, GABA enhances the reproduction and number of human β-cells and promotes the activation of growth and survival mechanisms by initiating PI3-K/Akt activation in β-cell islets [270]. Hosseini Dastgerdi et al. [271] found that administering GABA can decrease hepatic insulin resistance in pregnant diabetic rats and their offspring. This effect is achieved by modulating the insulin signaling and gluconeogenesis pathways. Administration of GABA to pregnant diabetic rats for 12 weeks resulted in a considerable reduction in plasma glucose levels in both the rats and their offspring. Another study found that empagliflozin and GABA, as the only treatment in streptozotocin induced diabetic mice, had beneficial effects on the preservation or growth of β-cell mass [272]. 

GABA-rich foods have been shown in several studies to have antidiabetic properties. In recent years, several clinical and animal studies have demonstrated the effectiveness of pre-germinated foods in improving DM. One contributing factor to their efficacy is the positive impact on GABA levels [273,274]. Research demonstrated notable improvements in administering GABA-rich-germinated adzuki beans to mice with type 2 DM. Specifically, fasting blood glucose levels decreased and there were significant enhancements in HOMA-β and HOMA-IR scores [275]. Li et al. [121] treated mice with streptozotocin-induced type 2 DM with GABA-rich yogurt and observed that this increased blood insulin levels, HOMA-β (a measure of β-cell activity), and better insulin sensitivity. C57BL/6J mice produced by a high-fat diet + streptozotocin (STZ) showed a drop in fasting blood glucose levels and improved glycolipid metabolism when supplemented with GABA-enriched germinated adzuki bean [276]. It was reported that GABA-rich fermented camel milk produced by *Levilactobacillus brevis* showed hypoglycaemic activity and decreased postprandial blood glucose levels in STZ-induced C57BL/6J mice [277]. Additionally, it has been suggested that the production of GABA by LAB strains has a noticeable impact on lowering glucose and insulin levels in the bloodstream during in vivo trials. This suggests there is potential for using GABA in pharmaceutical and food applications to decrease the occurrence of type 1 DM [278]. Furthermore, GABA-enriched fermented foods may regulate blood glucose levels in rats with type 2 DM by decreasing the activity of antioxidant enzymes, including glutathione, catalase, and superoxide dismutase. In the short term (6 weeks), fecal microbiota transplantation from healthy donors can successfully alleviate peripheral insulin resistance in patients, as evidenced by decreased HbA1c and increased plasma GABA [279]. Researchers are currently seeking safer and more efficient alternatives for the treatment of DM, considering its growing global prevalence and the adverse effects of current treatments. The human gut microbiota serves as a potent reservoir of bacteria that produce GABA. Hence, investigating the modulation of gut bacteria that produce GABA has great promise for future study. Although more research both in vitro and in vivo has shown that GABA has anti-diabetic properties, there is presently inadequate clinical data to support the use of GABA or GABA-rich diets in the treatment of DM. As a result, more clinical research is necessary to confirm the potential of GABA as an anti-diabetic agent and explore the transplantation of GABA-producing gut microbiota into diabetic patients.

### 4.6. Immunoregulatory Effects

Inflammation is the immune system’s response to external stressors, including physical injury, UV irradiation, microbial invasion, and immunological responses, etc. [280]. Inflammation has been associated with the synthesizing of many proinflammatory agents, including cytokines, NO, PGE2, and TNF-α. The prevalence of autoimmune diseases and immune response-related conditions, such as DM type I, atherosclerosis, and obesity, is on the rise. However, there is a scarcity of novel therapeutic strategies for these ailments. A growing body of research has examined the immunomodulatory function of microorganisms since it was discovered that gut microbiota protects immunological homeostasis. The immunological activities of the host are regulated by GABA, which LABs produce. GABA has been shown as an anti-inflammatory agent, as it inhibits the synthesis of proinflammatory mediators and improves symptoms associated with inflammation. GABA acts as a neurotransmitter inhibitor and significantly modulates the immune system [13,24]. Han et al. [281] found that GABA had anti-inflammatory activity by suppressing the synthesis and expression of iNOS, IL-1β, and TNF-α in RAW 264.7 cells treated with LPS. GABA improved the reduction of overall healing duration and promoted early wound healing. It has been shown that in LPS-induced mouse macrophage RAW 264.7 cells, NO generation and NO synthase expression are suppressed by GABA-enriched sea tangle L. japonica extract [282]. GABA-rich germinated brown rice reduced the release of IL-8, MCP-1, and ROS from Caco-2 human intestinal cells stimulated by IL-1 and H_2_O_2_ [283].

GABA-rich fermented *Aronia melanocarpa* extract was found to have anti-inflammatory properties through immune response modulation in Balb/c mice and inhibition of proinflammatory cytokines in RAW 264.7 cells. The findings from the in vivo experiments demonstrated that consuming fermented *Aronia melanocarpa* extract, which is rich in GABA, significantly impacted various immune parameters. Specifically, oral administration of doses (125, 250, and 500 mg/kg body weight) for 21 days resulted in enhanced proliferation of splenocytes and lymphocytes. Moreover, there was an increase in the expression of CD4^+^ and CD8^+^ T-cells, while the levels of TNF-α and IL-6 were reduced in a dose-dependent manner [284]. Likewise, the anti-inflammatory properties of GABA-enriched fermented strawberry juice were assessed, and findings revealed that COX-2 gene expression in LPS-stimulated RAW 264.7 macrophages reduced TNF-α, IL-6, and CXCL1 levels in mice given intraperitoneal LPS [141]. The GABA-rich extract derived from the red microalgae *Rhodosorus marinus* has been found to have an adverse effect on the expression and release of the proinflammatory cytokine IL-1α in normal human keratinocytes that have been stimulated with phorbol myristate acetate. This suggests the extract can treat sensitive skin, atopy, and dermatitis [285].

**Table 4:** In vitro and in vivo studies of the effects of GABA produced by LAB.

Author (Ref.)	Study Design	Foods	Micro-Organism	Model and Dosage	Outcomes
**Neuroprotection**					
Reid et al. (2018) [177]	In vivo	Gaba-enriched fermented sea tangle (*Laminaria japonica*)	*Levilactobacillus brevis* BJ20	Scopolamine-and ethanol-induced dementia model mice, 4 weeks	49.5 g/100 g, GABA ○ reversed cognitive impairment ○ reversed neuroplastic dysfunction
Seo et al. (2012) [173]	In vitro In vivo	Kimchi	*Lactobacillus sakei* B2-16	Reduced cognitive function mouse model with scopolamine and PC-12 cells, 24 h	46.69 mg/mL GABA ○ enhanced memory recovery, ○ increased neurite growth ○ increased neurite differentiation
Li et al. (2016) [150]	In vitro	Fermented chickpea milk (Kabuli)	*Lactiplantibacillus plantarum*	Noroendokrin MnCl_2_ induced PC12 cells, 30 min	537.23 mg/L GABA ○ protected the PC12 cells against MnCl_2_-induced injury
Cho et al. (2007) [50]	In vitro	Kimchi	*Lactobacillus buchneri*	100 g/mL, neuronal cells, 24 h	251 mM with a 94% GABA conversion rate ○ protected neuronal cells against neurotoxicant-induced cell death.
Reid et al. (2018) [176]	Human	Gaba-enriched fermented sea tangle (Laminaria japonica)	*Levilactobacillus brevis* BJ20	60 moderately active elderly subjects, randomized, double-blind, and placebo-controlled study, GABA-enriched fermented sea tangle for 6 weeks	1.5 g/d GABA-enriched fermented sea tangle ○ improved neuropsychological test scores ○ increased antioxidant activity of GPx, GSR, and SOD
Choi et al. (2016) [184]	Human	Gaba-enriched fermented sea tangle (Laminaria japonica)	*Levilactobacillus brevis* BJ20	21 middle-aged female subjects randomized, double-blind, placebo-controlled study, GABA-enriched fermented sea tangle for 8 weeks	1000 mg/d GABA-enriched fermented sea tangle ○ increased serum brain-derived neurotrophic factor level that associated with a lower risk for dementia and Alzheimer’s disease
**Anti-hypertension**					
Zareian et al. (2015) [199]	In vivo	Wheat-based fermented rice	*Lactiplantibacillus plantarum* MNZ	Spontaneously hypertensive rats, diet with fermented rice for 10 weeks	115.2 mg/kg GABA ○ decreased the systolic blood pressure ○ improved aortic endothelin-1 protein, plasma norepinephrine, and superoxide dismutase activity
Tsai et al. (2013) [205]	In vivo	Gaba-enriched Chingshey purple sweet potato-fermented milk	*Lactobacillus gasseri* BCRC 14619	Spontaneously hypertensive rats, a 2.5 mL dose of fermented milk for 5 weeks	2.5-mL Chingshey purple sweet potato fermented milk (600 μg GABA/mL) ○ decreased both systolic blood pressure ○ decreased diastolic blood pressure
Lin et al. (2012) [206]	In vivo	Gaba from probiotic-fermented purple sweet potato yogurt	*Lactobacillus acidophilus* BCRC 14065 *Lactobacillus delbrueckii* ssp. *lactis* BCRC 12256	Spontaneously hypertensive rats, GABA from probiotic-fermented purple sweet potato yogurt for 8 weeks	150 µg/2.5 mL (10%) and 1500 µg/2.5 mL/kg (100%) and GABA from probiotic-fermented purple sweet potato yogurt ○ decreased abnormal myocardial architecture and enlarged interstitial spaces at both doses ○ prevented the progression of cardiac hypertrophy at both doses
Liu et al. (2011) [194]	In vivo	Fermented milk	*Lactobacillus paracasei* subsp. NTU 101 *Lactiplantibacillus plantarum* NTU 102	Spontaneously hypertensive rats, fermented milk for 8 weeks	1.36 mg/kg BW/day ○ decreased systolic and diastolic blood pressures ○ reduced the disorganization of the media layer of aortic tissue
Abd El-Fattah et al. (2018) [192]	In vitro	Fermented milk	*Lactobacillus helveticus* *Lacticaseibacillus rhamnosus*	Spectrophotometry	ACE inhibitory activity, 88 %
Jang et al. (2015) [208]	In vitro	Soybean	*Levilactobacillus brevis*	In vitro ACE-inhibitory activity determination	1.9 g/kg GABA ○ exhibited higher ACE inhibitory activity
Torino et al. (2013) [211]	In vitro	Fermented lentils	*Lactiplantibacillus plantarum*	In vitro ACE-inhibitory activity determination	0.42 mg/g extract ○ ACE inhibitory activity
Nejati et al. (2013) [190]	In vitro	Fermented milk	*Lactococcus lactis* DIBCA2 *Lactiplantibacillus plantarum* PU11	In vitro ACE-inhibitory activity determination	77.4 mg/kg GABA (produced from Lactiplantibacillus *plantarum* PU11), 144.5 mg/kg (produced from *Lactococcus lactis* DIBCA2 and Lactiplantibacillus *plantarum PU11*) ○ ACE-inhibitory activity (IC50 = 0.70 ± 0.07 mg/mL) and a concentration of GABA (ca. 144.5 mg/kg)
Sun et al. (2009) [189]	In vitro	Fermented milk	*Lactobacillus helveticus*	Response surface methodology	ACE inhibitory activity higher than 50%
Becerra-Tomás (2015) [207]	Human	Gaba-rich bread	*Levilactobacillus brevis* CECT 8183	30 subjects patients with pre or mild-to-moderate hypertension, randomized, double-blind, crossover study, GABA-rich bread for 12 weeks	120 g/day bread (22.8 mg/100 g of GABA) ○ decreased diastolic blood pressure at rest ○ decreased 24-h ambulatory blood pressure
Pouliot-Mathieu et al. (2013) [196]	Human	Cheddar cheese	*Lactococcus lactis*	23 adult male subjects with slightly elevated blood pressure, cheddar cheese for 12 weeks	50 g of Cheddar cheese (16 mg GABA) ○ decreased systolic pressure ○ decreased mean blood pressure
**Anti-insomnia and anti-depression**					
Wu et al. (2021) [138]	In vivo	Adzuki bean sprout fermented milk	*Levilactobacillus brevis* J1 *Lactobacillus bulgaricus* *Lactiplantibacillus plantarum*	Male mouse model of mild depression exposed to social frustration stress for 10 days	3.43 mg/kg GABA ○ reduced mild depression-like symptoms ○ increased social interaction ○ enhanced the pleasure derived from movement ○ increased dopamine in the hippocampus
Yu et al. (2020) [120]	In vivo	Fermented milk	*Levilactobacillus brevis*	Male ICR mice, fermented milk for 30 days	33.33 mg/kg b.w. GABA ○ decreased in anxiety behavior
Ko et al. (2013) [234]	In vivo	Black soybean milk	*Levilactobacillus brevis* FPA 3709	Forced swimming rat model, black soybean milk for 6 weeks	35 mg/kg b.w. including 2.5 mg GABA/kg b.w., and 70 mg/kg b.w. including 5.0 mg GABA/kg b.w. ○ both dosages showed periods of inactivity similar to the effect of the antidepressant drug
Byun et al. (2018) [286]	Human	Fermented rice	*Lactobacillus sakei* B2-16	Adult subjects with insomnia symptoms, 4 weeks	300 mg of GABA produced from fermented rice (tablet form) ○ decreased the sleep latency ○ increased the sleep efficacy
Nakamura et al. (2009) [239]	Human	GABA-enriched Chocolate	*Lactobacillus hilgardii* K-3	Healthy male subjects, 15 min	10 g chocolate enriched with 28 mg GABA ○ improvement in heart rate variability from a stressful to a normal state
**Anti-diabetic**					
Zhang et al. (2022) [276]	In vivo	GABA-enriched çimlendirilmiş adzuki fasulyesi	Germination	HFD+STZ-induced C57BL/6J male mice, GABA-enriched germinated adzuki beans for 6 weeks	0.1 g GABA/kg diet/day ○ decreased fasting blood glucose ○ improved glycolipid metabolism
Abdelazez et al. (2022) [277]	In vivo	Fermented camel milk	*Levilactobacillus brevis* KLDS_1.0727_ or KLDS_1.0373_ strains	STZ-induced C57BL/6J mice, Lactobacillus brevis fermented camel milk for 4 weeks	GABA postbiotic produced by *Levilactobacillus brevis* demonstrated hypoglycemic activity and lowered postprandial blood glucose levels
Jeong et al. (2021) [287]	In vivo	GABA-enriched fermented noodles	*Bacillus subtilis*	HFD+STZ-induced mice, 300 mg/kg noodles with fermented lettuce extract	In diabetic mice, enriched GABA-fermented noodles increased insulin resistance and glucose tolerance
Jiang et al. (2021) [275]	In vivo	GABA-enriched germinated adzuki beans	Germination	HFD+STZ-induced mice, GABA-enriched germinated adzuki beans for 6 weeks	35 g GABA-enriched germinated adzuki bean treated groups ○ decreased fastin serum glucose ○ improved HOMA-β and HOMA-IR
Li et al. (2020) [121]	In vivo	GABA-enriched yogurt	*Streptococcus thermophilus*	HFD+STZ-induced type 2 DM C57BL/6 mice, drinking water containing 0.5–2 g/L GABA-rich yogurt for 12 weeks	2 g/L GABA yogurt ○ improved insulin sensitivity ○ increased serum insulin ○ regulated HOMA-β ○ improved islet cell morphology
Chung et al. (2019) [288]	In vivo	GABA-enriched Keunnunjami powder	Germination	Female ovariectomized Sprague-Dawley rats, GABA-enriched Keunnunjami powder for 8 weeks	Reducing blood glucose and plasma insulin levels, adipokine concentrations, and hepatic glucose-regulating enzyme activity
Pae et al. (2022) [289]	In vitro	-	-	Islet cell, 100 μM GABA	GABA elevated intracellular calcium levels in pancreatic β-cells, resulting in the depolarization of the cell membrane
Ghani et al. (2019) [290]	In vitro	-	-	Rat pancreatic ductal epithelial-like stem cells, 5–5000 μM GABA	Significantly elevated the concentration of insulin in the cell clusters
Rancourt-Bouchard et al. (2020) [291]	In vivo	GABA-enriched cheddar cheese	*Lactococcus lactis* ssp. *Lactis*	55 healthy men and women (1) no dairy (control diet) (2) 3 daily servings of 1% fat milk (3) 1 daily serving of 31% fat cheddar cheese naturally enriched in GABA for 6 weeks	There was no significant difference between all diets for markers of glucose/insulin homeostasis
**Anti-Inflammatory**					
Weerawatanakorn et al. (2023) [292]	In vivo	GABA-fortified oolong tea	-	HFD-induced obesity male C57BL/6J mice - chow diet - HFD - HFD+ GABA-fortified oolong tea	GABA-fortified oolong tea s reduced leptin expression in epididymal adipose tissue and showed a protective effect on nonalcoholic fatty liver disease. It boosted lipid metabolism and promoted fatty acid oxidation. It also reduced lipogenesis-related protein levels of sterol regulatory element binding protein, acetyl-CoAcarboxylase, and fatty acid synthase and inhibited hepatic triglyceride levels.
Lee et al. (2022) [293]	In vivo	GABA-enriched salt	*Levilactobacillus brevis*	Cisplatin-induced nephrotoxicity in mice were administered 92.69/111.92/97.25 mg/g GABA salt/lacto GABA salt/postbiotics GABA salt	Reduced expression levels of HMGB-1, proinflammatory mediators, CoX-2, IL-1β, and TNF-α
Ali et al. (2021) [284]	In vivo In vitro	GABA-rich fermented Aronia melanocarpa extract	*Lactiplantibacillus plantarum*	Female BALB/c mice were administered 125, 250, and 500 mg/kg of Aronia melanocarpa extract for 21 days RAW 264.7 cells were treatment Aronia melanocarpa extract for 30 min	GABA-rich fermented Aronia melanocarpa extract stimulated the immune system in mice and inhibited proinflammatory cytokines in RAW 264.7 cells to provide anti-inflammatory effects
Cataldo et al. (2020) [141]	In vivo In vitro	GABA-enriched fermented strawberry juice	*Levilactobacillus brevis*	Male Balb/c mice were treated with GABA-enriched fermented strawberry juice (~140 mM GABA) or the diluted GABA-enriched fermented strawberry juice (~70 mM GABA) RAW 264.7 macrophages were treatment GABA-enriched fermented strawberry juice (0.1 or 1 mM GABA)	GABA-enriched fermented strawberry juice was capable of reducing peritoneal, intestinal, and serum TNF-α, IL-6, and CXCL1 levels while increasing IL-10 and IFN-γ. The GABA-enriched fermented strawberry juice exhibited a notable capacity to substantially decrease the expression of the CoX-2 gene in RAW 264.7 macrophages.
Zheng et al. (2023) [294]	In vitro	GABA-enriched Moringa oleifera leaves	*Lactiplantibacillus plantarum* LK-1	RAW 264.7 cells	GABA-enriched Moringa oleifera leaves could effectively alleviate the LPS-induced inflammatory response by inhibiting the secretion of proinflammatory cytokines via TLR-4/NF-kB inflammatory signaling pathway inhibition.
Ngo et al. (2022) [295]	In vitro	GABA-enriched rice bran	*Limosilactobacillus fermentum*	RAW 264.7 cells	GABA-enriched rice bran was found to suppress the levels of inducible NO synthase and CoX-2 enzymes.
Bajić et al. (2020) [296]	In vitro	-	*Levilactobacillus brevis*	Mesenteric lymph node cells	The GABA produced by this strain showed inhibitory effects on the proliferation of mesenteric lymph node cells, as well as the production of IFN-γ and IL-17. Additionally, it reduced the expression of proinflammatory markers such as MHCII and CD80. The supernatants containing GABA showed the most potent stimulating effects on the production of immunoregulatory molecules, including Foxp3^+^, IL-10, TGF-β, CTLA4, and SIRP-α.
Sokovic Bajic et al. (2019) [297]	In vitro	-	*Levilactobacillus brevis*	Caco-2 cells	The anti-inflammatory effects of GABA-producing *Levilactobacillus brevis* were observed in reducing IL-1β-induced inflammation and promoting the expression of tight junction proteins and TGF-β cytokine.

## 5. Conclusions

Some metabolites of LABs, which are the most commonly used microorganisms in the food industry, can also be used for similar purposes to improve food safety and quality. GABA, one of these metabolites known to be synthesized at different levels by many LABs, helps improve the taste, flavor, aroma, and texture of the food it contains, and increases protein digestibility. Moreover, it can extend the shelf life of foods by preventing the proliferation of pathogenic microorganisms. Foods with increased GABA content can be produced as a result of the fermentation of meat, grains, milk, fruits, vegetables, and legumes with LABs. With the demonstration of these advantages they provide to foods, the use of GABA-producing LABs in the food industry has attracted great attention in recent years. However, it should not be ignored that some microorganisms may cause spoilage in foods, and strains with high GABA efficiency and proven safety should be preferred. 

The positive effects of GABA, produced by LABs and called postbiotic, on neuroprotection, improving sleep quality, alleviating depression, and relieving pain have accelerated efforts to increase GABA production. Moreover, considering the various health benefits of GABA-enriched foods such as antidiabetics, antihypertension, and anti-inflammatory, their use will become more widespread in the coming years. 

On the other hand, it should not be ignored that during the fermentation of foods with some LABs, some toxic biogenic amines such as histamine and tyramine, which can have negative effects on health, may be produced. For this reason, more comprehensive studies involving in vitro and in vivo analyses and animal and human subjects should be conducted to identify LAB strains that do not form biogenic amines, have high GABA yield, and are safe. Moreover, the oral intake of GABA theoretically could cause food–drug interactions by increasing the sedative effect of barbiturates or benzodiazepines. On the other hand, GABA intake can alleviate the symptoms of barbiturate withdrawal. Also, it is important to mention that GABA intake through nutrition can regulate the lateral hypothalamus (the so-called hunger center) and thereby could positively influence obesity and overeating disorders.

In summary, this review draws attention to the synthesis of GABA produced from LABs, the factors affecting its synthesis, and efforts to improve GABA production, underlines its areas of use in the food industry and the benefits it provides to the foods it contains. Thus, it is predicted that this review article will pave the way toward serving for future studies on increasing GABA production and developing GABA-rich functional products.

## Data Availability

No new data were created or analyzed in this study. Data sharing is not applicable to this article.
